# Lysosomal enzyme cathepsin D protects against alpha-synuclein aggregation and toxicity

**DOI:** 10.1186/1756-6606-1-17

**Published:** 2008-11-21

**Authors:** Liyan Qiao, Shusei Hamamichi, Kim A Caldwell, Guy A Caldwell, Talene A Yacoubian, Scott Wilson, Zuo-Lei Xie, Lisa D Speake, Rachael Parks, Donna Crabtree, Qiuli Liang, Stephen Crimmins, Lonnie Schneider, Yasuo Uchiyama, Takeshi Iwatsubo, Yi Zhou, Lisheng Peng, YouMing Lu, David G Standaert, Ken C Walls, John J Shacka, Kevin A Roth, Jianhua Zhang

**Affiliations:** 1 Department of Pathology, University of Alabama at Birmingham, Birmingham, USA; 2 Department of Biological Sciences, The University of Alabama, Tuscaloosa, USA; 3 Department of Neurobiology, University of Alabama at Birmingham, Birmingham, USA; 4 Department of Neurology, University of Alabama at Birmingham, Birmingham, USA; 5 Center for Neurodegeneration and Experimental Therapeutics, University of Alabama at Birmingham, Birmingham, USA; 6 Department of Cell Biology and Neurosciences, Osaka University, Osaka, Japan; 7 Department of Neuropathology, Graduate School of Medicine, Department of Neuropathology and Neuroscience, Graduate School of Pharmaceutical Sciences, University of Tokyo, Tokyo, Japan; 8 Biomolecular Science Center, Burnett College of Biomedical Sciences, Orlando, USA; 9 Department of Veterans Affairs, Birmingham VA Medical Center, Birmingham, AL35294, USA

## Abstract

α-synuclein (α-syn) is a main component of Lewy bodies (LB) that occur in many neurodegenerative diseases, including Parkinson's disease (PD), dementia with LB (DLB) and multi-system atrophy. α-syn mutations or amplifications are responsible for a subset of autosomal dominant familial PD cases, and overexpression causes neurodegeneration and motor disturbances in animals. To investigate mechanisms for α-syn accumulation and toxicity, we studied a mouse model of lysosomal enzyme cathepsin D (CD) deficiency, and found extensive accumulation of endogenous α-syn in neurons without overabundance of α-syn mRNA. In addition to impaired macroautophagy, CD deficiency reduced proteasome activity, suggesting an essential role for lysosomal CD function in regulating multiple proteolytic pathways that are important for α-syn metabolism. Conversely, CD overexpression reduces α-syn aggregation and is neuroprotective against α-syn overexpression-induced cell death in vitro. In a *C. elegans *model, CD deficiency exacerbates α-syn accumulation while its overexpression is protective against α-syn-induced dopaminergic neurodegeneration. Mutated CD with diminished enzymatic activity or overexpression of cathepsins B (CB) or L (CL) is not protective in the worm model, indicating a unique requirement for enzymatically active CD. Our data identify a conserved CD function in α-syn degradation and identify CD as a novel target for LB disease therapeutics.

## Introduction

Patients with α-synuclein (α-syn) A53T, A30P, E46K mutations or gene amplification develop typical Parkinson's disease (PD) and often an associated dementia[1]. However, in > 90% of PD cases, and almost all dementia with Lewy body (DLB) and Lewy body variant of Alzheimer's disease (LBVAD) cases, α-syn aggregation occurs yet without a clear evidence of mutation or up-regulation of α-syn mRNA transcription. Therefore, impaired α-syn clearance may play a more important role than α-syn overexpression in neuronal α-syn accumulation and neurodegenerative disease pathogenesis.

Experiments in vitro have shown that α-syn can be cleared by the cytosolic ubiquitin-proteasome system (UPS), and/or lysosome-mediated autophagic pathways[2]. The UPS degrades short-lived, misfolded and/or damaged proteins via an ubiquitin-dependent signaling pathway. Macroautophagy is initiated by de novo synthesis of double membrane vesicles in the cytoplasm. These vesicles encircle long-lived or damaged proteins or organelles by an unknown signaling mechanism and deliver these cargos to lysosomes for degradation. Chaperone-mediated autophagy (CMA) is initiated by chaperones binding to KFERQ-consensus sequence-containing cytosolic proteins followed by delivery to the lysosomes via lysosomal membrane LAMP-2a receptors. Wildtype α-syn has a pentapeptide sequence that can serve as a CMA recognition motif and can be translocated to the lysosome, while dopamine modified or pathogenic A53T and A30P mutant α-syn block CMA [3,4].

Lysosomal function declines with age in the human brain[5]. Accumulation of autophagic vacuoles (AVs) has been reproducibly observed in postmortem AD and PD patient brains compared to normal controls, consistent with either an overproduction of AVs or a deficit in autophagolysosomal clearance[6]. Enhancing macroautophagy by either mTOR-dependent or independent mechanisms can help clear aggregation-prone proteins, including huntingtin, A53T and A30P mutant α-syn [7,8]. However, because both macroautophagy and CMA are dependent on intact lysosomes, enhancing macroautophagy may not be effective in clearing potentially neurotoxic proteins if lysosomal function is impaired. Understanding the role of lysosomal enzymes in α-syn clearance may provide new strategies for LBVAD, DLB, and PD therapy.

Cathepsin D (CD) is the principal lysosomal aspartate protease and a main endopeptidase responsible for the degradation of long-lived proteins [9,10], including α-syn[11]. CD is expressed widely in the brain, including the cortex, hippocampus, striatum, and dopaminergic (DA) neurons of the substantia nigra (SNr)[12]. CD is synthesized as a precursor with a signal peptide cleaved upon its insertion into endoplasmic reticulum[13]. The CD zymogen is activated in an acidic environment by cleavage of the pro-peptide. CD is also up-regulated in AD brains as an early event, but whether CD up-regulation is coincidental, disease promoting, or compensatory is unclear[14].

CD gene *(Ctsd) *homozygous inactivation was reported to cause human congenital neuronal ceroid lipofuscinosis (NCL) with postnatal respiratory insufficiency, status epilepticus, and death within hours to weeks after birth[15]. These patients had severe neurological defects at birth and neuronal α-syn accumulation was not examined. Another patient with significant loss of CD enzymatic function (7.7% Vmax from patient fibroblast lysates compared to controls) due to compound heterozygous missense mutations developed childhood motor and visual disturbances, cerebral and cerebellar atrophy, and progressive psychomotor disability[16]. Conceivably, milder forms of CD deficiency could predispose to late onset neurodegenerative disorders, including AD and PD. Parkinsonism has been noted in lysosomal tripeptidyl peptidase I deficient patients[17], adult forms of NCL patients[18], and Gaucher disease patients[19]. α-syn aggregation has been reported in both neurons and glia in several lysosomal disorders, such as Gaucher disease[20], Niemann-Pick disease[21], GM2 gangliosidosis, Tay-Sachs, Sandhoff disease, metachromatic leukodystrophy, and beta-galactosialidosis[22].

Significant α-syn accumulation has not been previously reported in mouse models of proteolytic disorders involving proteasomes, autophagy or other lysosomal proteases [23-26]. Here we report a robust α-synucleinopathy in CD deficient mice, despite the compensatory up-regulation of other lysosomal proteases, and the absence of an increase of α-syn mRNA expression. We found that proteasome activities are significantly reduced in the CD-deficient brain, whereas several key UPS factors are either normal or up-regulated, indicating crosstalk between lysosomal and proteasomal activities at the levels of signaling rather than a reduction of protein levels. Finally, we demonstrate that CD, but not Cathepsin B (CB) or Cathepsin L (CL), overexpression reduces α-syn aggregation and provides potent neuroprotection from α-syn-induced neuron death in vitro and in vivo.

## Results

### CD deficient mice exhibit extensive accumulation of α-syn in neurons

**Figure 1:**
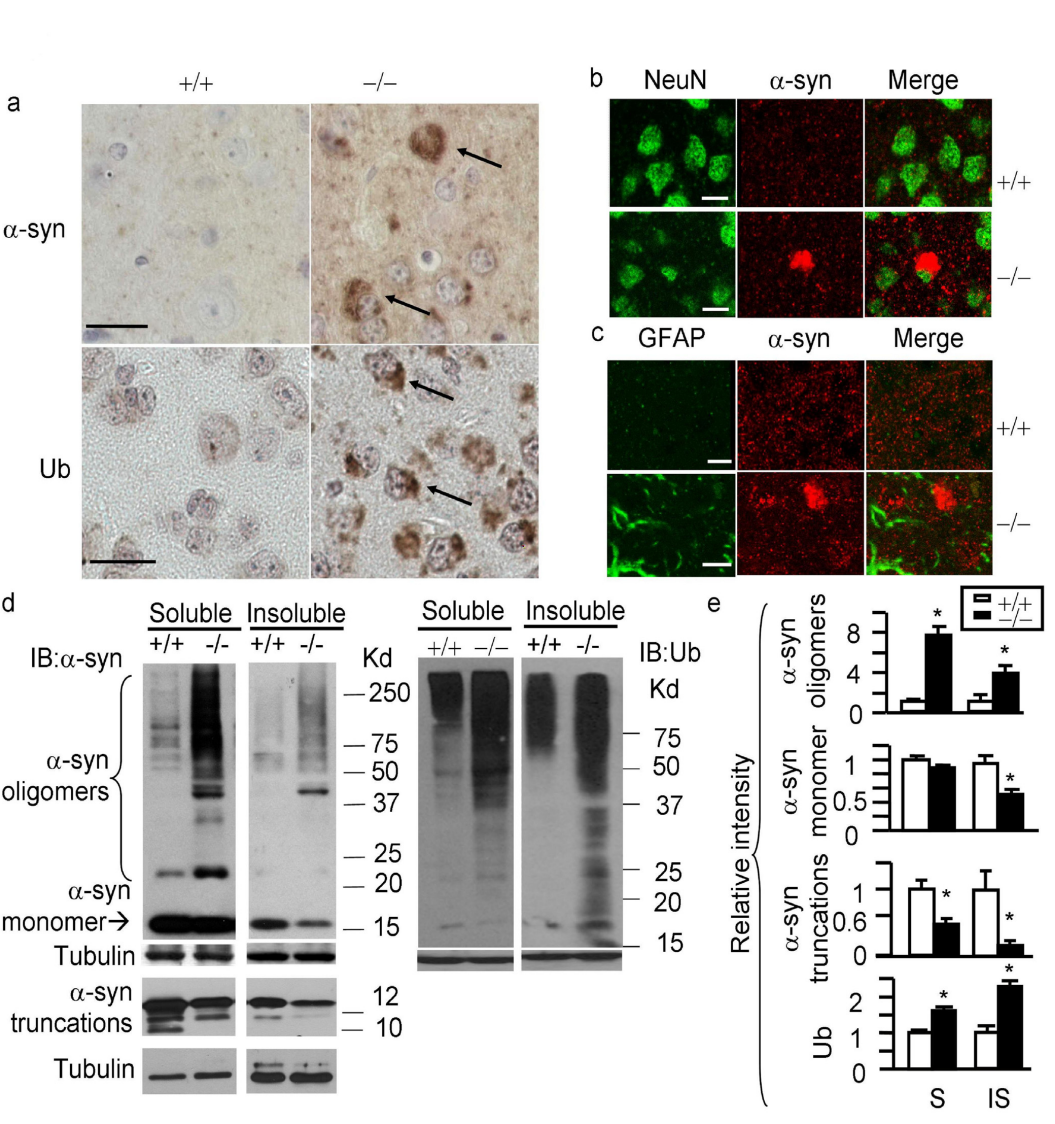
**α-syn accumulates in neuronal cell bodies in p25 *Ctsd*-/- cortex**. a. Immunohistochemical detection of α-syn and ubiquitin in p25 *Ctsd*+/+ (+/+) and *Ctsd*-/- (-/-) cortex. Scale bar = 20 micron. Arrows point to intense α-syn and ubiquitin immunoreactive cells. b. α-syn accumulation occurs in NeuN+ neuronal cell bodies. α-syn = Cy3 (red), NeuN = FITC (green). Wildtype (+/+) brains exhibit diffuse α-syn staining consistent with a synaptic distribution. *Ctsd*-/- brains showed neurons with cytoplasmic accumulation of α-syn immunoreactivity. Scale bar = 10 micron. c. α-syn does not exhibit pronounced accumulation in GFAP+ cells. α-syn = Cy3 (red). GFAP = FITC (green). Scale bar = 10 micron. d. Accumulation of high molecular weight α-syn and ubiquitinated proteins in both the TritonX-100 soluble and the insoluble fractions of the *Ctsd*-/- mice. Intensity of α-syn monomer, α-syn oligomers, and Ub-positive smears were quantified and compared between *Ctsd*+/+ and *Ctsd*-/- extracts. Truncated 12 kDa and 10 kDa α-syn fragments are reduced in *Ctsd*-/- extracts. e. Quantification of the western results. n = 3 mice each genotype. *p < 0.05 compared to *Ctsd*+/+ by Student t-test. S = TritonX-100 soluble. IS = TritonX-100 insoluble. f. A small fraction of accumulated α-syn in *Ctsd*-/- brains being mono-ubiquitinated. Western blot analysis of TritonX-100 soluble *Ctsd*-/- brain extract (Input), pulldown product by a polyclonal α-syn antibody C-20 (Santa Cruz) (middle lane), and pulldown product by a same-isotype control antibody (IgG). Immunoblot was probed with mAb1510 against ubiquitin. Shown are the 25 kDa bands of ubiquitin immunoreactivity that were pulled down by the C-20 anti-α-syn antibody. g. Western blot analysis of ubiquitin with p21 *Ctsd*+/+ and *Ctsd*-/- cortical extracts. The intensity of each lane was quantified and shown in the bar graph. *p < 0.05 Student t-test. h. Western blot analyses of *Ctsd*+/+ and *Ctsd*-/- cortical extracts, together with human DLB brain extracts using anti-phospho-α-syn antibody provided by Dr. Iwatsubo [33,36,38]. Arrows indicate positions of immunoreactive bands that are increased in *Ctsd*-/- mice and the position of α-syn monomer.

To investigate the involvement of lysosomal functions in α-syn clearance, we analyzed mice deficient in CD, previously generated by a targeted insertion of the *neo *marker in exon 4[27]. CD-deficient (*Ctsd*-/-) mice die at approximately postnatal day 26 (p26) due to a combination of nervous system and systemic abnormalities. Extensive neuron death resulting from activation of both apoptotic and non-apoptotic pathways has been observed in these mice [27-31]. We examined brains from p21 and p25 *Ctsd*-/- mice and found significant α-syn accumulation in neuronal cell bodies in p25 *Ctsd*-/- but not wildtype cortex (Fig. 1a). In contrast to the brains of human lipidoses patients[32] where α-syn aggregates are found in both neurons and glia and co-localize with lipids, in *Ctsd*-/- brains α-syn accumulations do not co-localize with autofluorescent lipofuscin (data not shown).

Furthermore, α-syn accumulations in *Ctsd*-/- brains were present in cells co-expressing the neuron marker, neuronal nuclear antigen (NeuN), but not in glial fibrillary acidic protein (GFAP)-immunoreactive astrocytes (Fig. 1b–c). Accumulation of ubiquitinated proteins also occurs in *Ctsd*-/- cortex (Fig. 1a). A relatively small fraction of neurons (~5%) with intense accumulated α-syn immunostaining also exhibit co-localization with intense accumulated ubiquitin staining, consistent with the observation that a small fraction of α-syn in LB is ubiquitinated [33-36]. Correspondingly, we found elevated levels of high molecular weight but not monomeric α-syn, and high molecular weight ubiquitinated proteins in both TritonX-100 soluble and insoluble extracts from the cortex of *Ctsd*-/- mice by western blot analyses compared to *Ctsd*+/+ mice, similar to what occurs in LB diseases (Fig. 1d–e). Truncated 12 kDa and 10 kDa α-syn fragments are reduced in *Ctsd*-/- extracts (Fig. 1d–e).

Antibody pulldown experiments indicate that a small fraction of accumulated α-syn in *Ctsd*-/- brains is mono-ubiquitinated (Fig. 1f). These results are consistent with a small fraction of accumulated α-syn in *Ctsd*-/- brains being mono-ubiquitinated [33-36]. Ubiquitin immunostaining is present in nearly all neurons with variable intensity. 5% of those neurons with intense accumulated ubiquitin staining also exhibit intense accumulated α-syn immunostaining in *Ctsd*-/- brain sections. Conversely, 5% of neurons with intense accumulated α-syn immunostaining also exhibit intense accumulated ubiquitin staining. p21 *Ctsd*+/+ and *Ctsd*-/- brains do not exhibit considerable difference in α-syn immunostaining intensity or protein level by western blot analyses. However, ubiquitin accumulation occurs as early as p21 in *Ctsd*-/- cortical extracts compared to wildtype cortical extracts (Fig. 1g).

Immunostaining with synphilin antibodies have not detected "aggregation-like" structures in the cell body where α-syn is. Nor did we observe increased synphilin in western blot analyses. Thus the α-syn accumulation is unlikely to be induced by synphilin accumulation in *Ctsd*-/- brains. The cytoplasmic microtubule-associated protein, tau, did not accumulate in *Ctsd*-/- cortex at p25 compared to wildtype cortex at p25 (data not shown), suggesting that CD deficiency does not have a general effect on the accumulation of all cytoplasmic proteins.

In human LB, 95% of the insoluble α-syn is phosphorylated [33-35,37,38]. We found that anti-phospho-α-syn antibody (from Dr. Iwatsubo [33,36,38]) did not recognize the intensely immunoreactive accumulated α-syn in *Ctsd*-/- brains, although numerous neurons in both wildtype and *Ctsd*-/- brains exhibited labeling with this antibody. Whether this antibody lacks specificity when used in mouse brain sections or the accumulated α-syn in *Ctsd*-/- brain is simply not phosphorylated cannot be determined from this immunohistochemical study. Western blot analyses indicate that a few immunoreactive bands are increased in *Ctsd*-/- extracts compared to wildtype extracts.

### Intense α-syn immunoreactive accumulations are outside of autophagosomes and lysosomes in affected neurons

**Figure 2:**
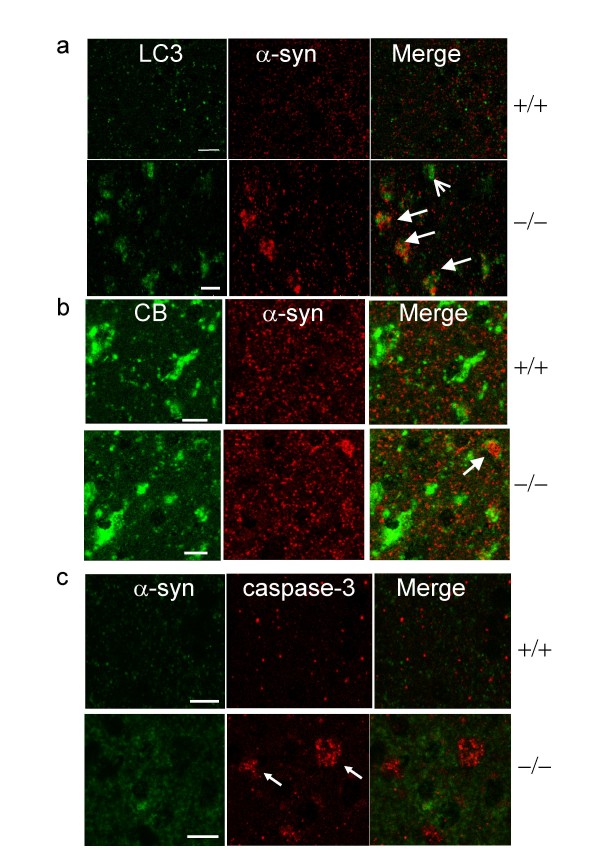
**α-syn accumulation does not co-localize with LC3 or CB immunoreactivity and is independent of active caspase-3 immunoreactivity**. a. Immunostaining with Atg8/LC3 and α-syn antibodies shows that LC3 staining is increased in *Ctsd*-/- mice compared to *Ctsd*+/+ mice, and intense α-syn immunoreactivity does not colocalize with LC3 immunoreactivity (arrows, p25). LC3 = FITC (green). α-syn = Cy3 (red). Arrowheads point to cells with high LC3 immunoreactivity but without intense α-syn immunostaining. b. α-syn accumulation does not overlap with CB immunoreactivity. CB = FITC (green). α-syn = Cy3 (red). Arrow points to α-syn intense immunoreactive staining adjacent to CB staining. c. Neurons with active caspase-3 immunoreactivity do not exhibit intense α-syn immunoreactivity in *Ctsd*-/- brains. α-syn = FITC (green). Active caspase-3 = Cy3 (red). Arrows point to active caspase-3 immunoreactivity. Scale bar = 10 micron. n = 3 mice each genotype.

Prior studies found autophagosomes start to accumulate in *Ctsd*-/- brains as early as p1, compared to *Ctsd*+/+ age-matched controls [28,30]. Furthermore, CB immunostaining as well as enzymatic activities are increased as early as p21 in *Ctsd*-/- brains [28,30]. Electron microscopy studies demonstrated that CB was associated with irregularly shaped and membrane-bound structures containing electron-dense materials, characterizing them as lysosomes in Ctsd-/- brains [28,30]. We found that α-syn accumulation does not become prominent until near p25 in *Ctsd*-/- brains. α-syn accumulation occurs in only a sub-population of neurons that exhibit enhanced Atg8 (AuTophaGy8)/LC3 (light chain 3) staining, indicating that AV accumulation precedes α-syn accumulation (Fig. 2a). We also found that α-syn accumulations are adjacent to, but do not overlap with, immunoreactivity for the AV-associated protein Atg8/LC3 or the lysosomal associated protein CB, suggesting that α-syn accumulation formed outside of autophagosomes and lysosomes (Fig. 2b). Neuronal populations immunoreactive for the apoptotic marker, cleaved caspase-3, are distinct from those with intense α-syn accumulation (Fig. 2c).

### α-syn accumulation is not due to up-regulation of its mRNA, and appears despite compensatory up-regulation of other proteases

**Figure 3:**
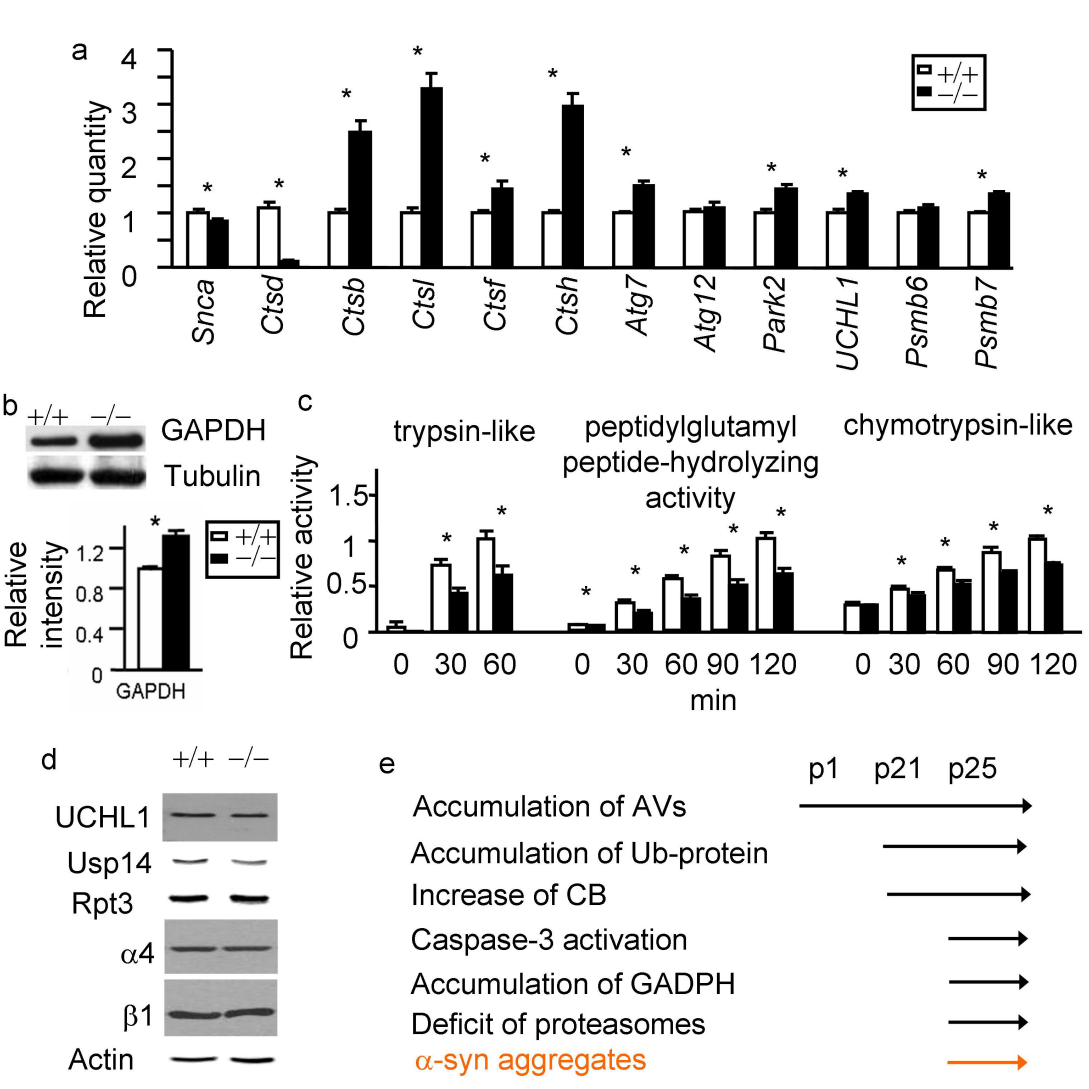
**Deficits of proteasomes in CD deficient mice**. a. α-syn (*Snca*) mRNA is down-regulated in CD deficient brains compared to wildtype control brains. *Ctsb, Ctsl, Ctsf, Ctsh, Atg7, UCHL1, Park2, and Psmb7 *mRNA levels are up-regulated. *Atg12 *and *Psmb6 *mRNA levels appear to be normal. b. Western blot analyses show an increase of steady state GAPDH, a CMA substrate. n = 3 p25 brain. *p < 0.05 by Student t-test, compared between *Ctsd*+/+ and *Ctsd*-/- brains. c. Extracts from *Ctsd*-/- cortex exhibit reduced proteasome activities compared to *Ctsd*+/+ as indicated by assays with trypsin-like fluorigenic substrate (VGR-AMC, reaching maximum at 60 min), chymotrypsin-like fluorigenic substrate (Z-GGL-AMC, reaching maximum at 120 min), and peptidylglutamyl peptide-like fluorigenic substrate (Suc-LLVY-AMC, reaching maximum at 120 min). The activities that are inhibitable by the proteasome inhibitor lactacystin were quantified. n = 3 mice each genotype. *p < 0.05 by Student t-test. d. Normal expression of proteins involved in UPS. Western blot analyses of UCHL1, Usp14, Rpt3, α4 and β1 indicate that these UPS factors are expressed normally in *Ctsd*+/+ and *Ctsd*-/- cortical extracts. Actin immunoblotting was used as a loading control. n = 3 mice each genotype. e. A diagram regarding onset of relative pathologies.

While bulk protein degradation appears to be normal in *Ctsd*-/- mice[27], we found that α-syn mRNA is down-regulated in *Ctsd*-/- brains at p25 when α-syn accumulation occurs (Fig. 3a). This is consistent with the finding that α-syn mRNA is either unchanged or down-regulated in the majority of sporadic PD cases[39], indicating that α-syn accumulation is unlikely to be the consequence of elevated gene expression. Prior studies reported that CB but not CL protein is up-regulated in *Ctsd*-/- brains at p23[28]. To better understand the role of CD in selective protein degradation and PD, we analyzed the expression of genes encoding other brain-enriched lysosomal proteases, autophagy-associated factors, proteasome subunits, and genes linked to familial PD. Interestingly, genes encoding other lysosomal cathepsins B, L, F and H (*Ctsb, Ctsl, Ctsf*, and *Ctsh*) mRNAs are all up-regulated at p25 (Fig. 3a). This result may suggest a common transcription regulatory mechanism for these cathepsins in response to CD deficiency. Alternatively, the influx of macrophages or microglia into the *Ctsd*-/- brain at this age may lead to an increase in cathepsin mRNA expression[40]. We also determined that accumulation of autophagosomes in *Ctsd*-/- neurons is accompanied with transcriptional up-regulation of *Atg7 *but not *Atg12 *(Fig. 3a). Both Atg7 and Atg12 are involved in an ubiquitin-like activity important for autophagosome expansion. Up-regulation of *Atg7 *may indicate an increase of autophagosome production in addition to a blockade of autophagy completion [28,38,40]. mRNA of *Park2 *which encodes Parkin, mutation of which has been found in a subset of autosomal recessive PD;*UCHL1*, mutation of which has been found in a subset of autosomal dominant PD; and *Psmb7 *which encodes proteasome 20S core β2 subunit are also modestly up-regulated in response to CD deficiency, indicating a compensatory response to CD deficiency at the level of gene transcription (Fig. 3a).

### CD deficiency reduces proteasome activities

In addition to deficient macroautophagy, we found an accumulation of GAPDH, a substrate of CMA (Fig. 3b). Interestingly, we also found reduced proteasome activity in *Ctsd*-/- brain extracts (Fig. 3c), suggesting a functional interaction between the two major α-syn clearance machineries, lysosomes and proteasomes. Accumulation of ubiquitinated proteins in *Ctsd*-/- brain appears at p21 compared to that in wildtype brains (Fig. 1g, 3e), when proteasome activities are unaltered as determined by the proteasome activity assay (data not shown). So far, none of the proteasome-related proteins we examined, including UCHL1, a gene mutated in familial PD and a ubiquitin hydrolase and E3 ligase; Usp14, a key deubiquitination enzyme; Rpt3, an ATPase regulatory subunit, a4 subunit that is important for the gating into the 20S core particle, and b1 subunit that is part of the proteasome core, were significantly changed as determined by western blot analyses in Ctsd-/- brains (Fig. 3d).

### Overexpression of CD reduces α-syn aggregation in mammalian cells

**Figure 4:**
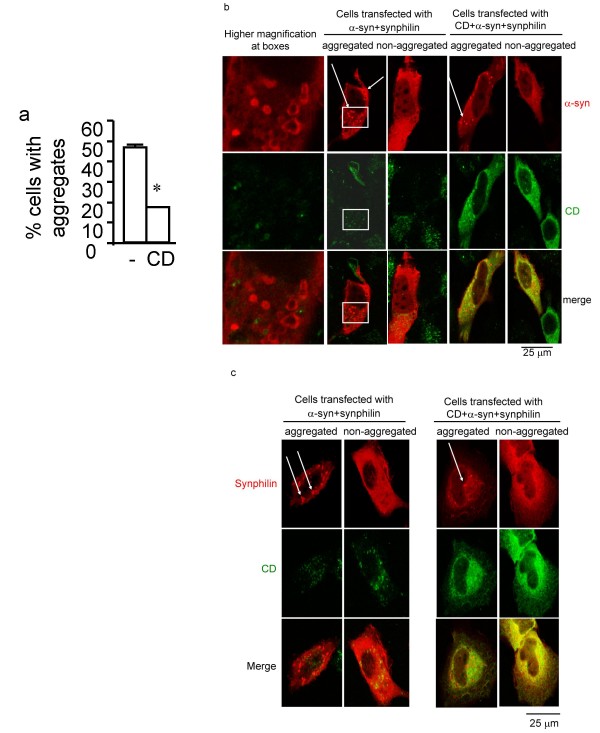
**CD reduces α-syn aggregation**. a. CD reduces α-syn aggregation in an aggregation assay. 50% of the cells transfected with α-syn-GFP, synphilin and empty vector exhibited visible α-syn aggregates. Co-transfection of CD together with α-syn-GFP and synphilin reduced the number of cells with visible α-syn aggregates to 20%. n = 3 independent transfection, each in quadruplicate. *p < 0.05 compared to absence of exogenous CD by Student t-test. b. Double immunostaining for CD and α-syn in the aggregation assay after transfection of H4 cells with α-syn+synphilin+vector and α-syn+synphilin+CD. Many more cells transfected with synphilin+α-syn+vector have α-syn aggregates than cells transfected with synphilin+α-syn+CD. α-syn = red; CD = green. Higher magnification images of the boxed areas are shown at the left-most panel. α-syn aggregates tended to be found in cellular areas with low levels of CD staining, in H4 cells with either α-syn+synphilin+vector or α-syn+synphilin+CD. Scale bar = 25 micron. Arrows point to α-syn aggregates. c. Double immunostaining for CD and the V5 tag of synphilin in the aggregation assay after transfection of H4 cells with α-syn+synphilin+vector and α-syn+synphilin+CD. Many more cells transfected with synphilin+α-syn+vector have α-syn aggregates than cells transfected with synphilin+α-syn+CD. Synphilin = red; CD = green. The levels of synphilin immunostaining were indistinguishable in cells transfected with synphilin+α-syn+vector versus cells transfected with synphilin+α-syn+CD. This is true in cells with or without aggregates. Scale bar = 25 micron. Arrows point to α-syn aggregates.

To further understand how CD activity influences α-syn homeostasis, we examined whether enhancing CD expression can reduce α-syn aggregation. We adopted the simple culture system developed by McLean and colleagues in which an α-syn-green fluorescent protein (GFP) fusion protein (α-syn-GFP) forms visible aggregates in cells when co-expressed with synphilin[41]. We transfected H4 neuroglioma cells with α-syn-GFP, synphilin, and CD. As a control, cells were transfected with α-syn-GFP, synphilin and empty vector pcDNA3.1. Approximately 50% of control transfected cells exhibited α-syn aggregates, consistent with published findings[41]. Remarkably, transfection of CD together with α-syn-GFP and synphilin led to less than 20% of transfected cells exhibiting α-syn aggregates (Fig. 4a). To address the issue of whether CD could affect synphilin levels, we performed immunocytochemistry experiments to compare α-syn+synphilin+vector transfected cells and α-syn+synphilin+CD transfected cells. We chose this approach because very low transfection efficiency was achievable (less than 5%) in this aggregation assay. Thus, we cannot perform western blot analyses or quantitative RT-PCR assays for this experiment. Double immunostaining for CD and the V5 epitope tag on synphilin and double immunostaining for CD and α-syn were performed (Figs. 4b and 4c). We quantified the number of transfected cells with α-syn aggregates and those without α-syn aggregates. Many more cells that were transfected with synphilin+α-syn+vector exhibited α-syn aggregates in comparison to cells transfected with synphilin+α-syn+CD. Nonetheless, the levels of synphilin immunostaining were indistinguishable in cells transfected with synphilin+α-syn+vector versus cells transfected with synphilin+α-syn+CD. This is true in cells with or without aggregates. We conclude that CD reduction of α-syn aggregation is unlikely to be secondary to a reduction in synphilin levels.

### Overexpression of CD is neuroprotective against α-syn toxicity in mammalian cells

**Figure 5:**
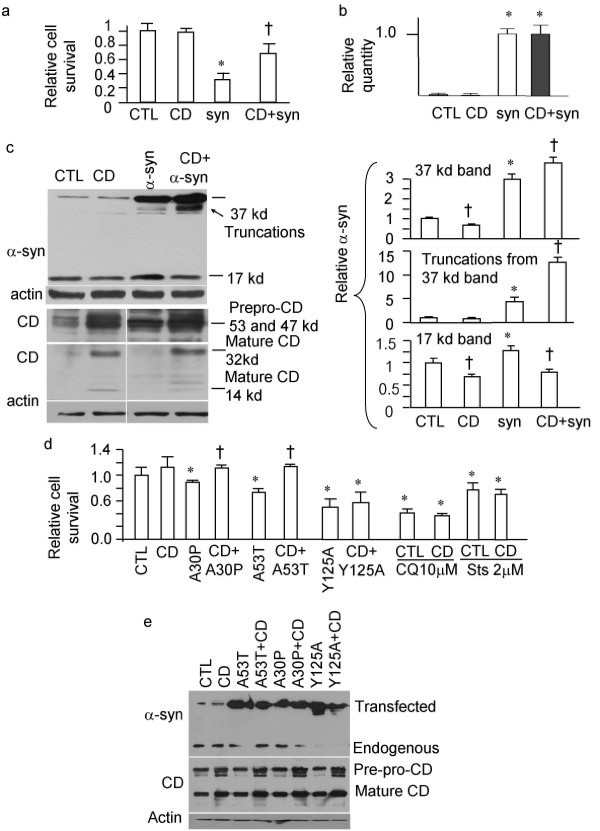
**CD reduces α-syn toxicity**. a. Enhanced CD expression protects against α-syn overexpression-induced cell death. GFP was visualized under the fluorescence microscope and demonstrated more survival cells after co-transfection of GFP-α-syn and CD compared to transfection with GFP-α-syn alone. Viable cells were counted by trypan blue exclusion method. b. mRNA of α-syn is unchanged by CD transfection. SHSY5Y cells were transfected with vector alone, CD, α-syn, or α-syn+CD. Paired Student t-tests were conducted on RQ values for each group to determine significance. c. Western blot analyses indicate that CD transfection results in truncation of α-syn-GFP (the appearance of a band below the full-length 37 kDa band), and a reduction of endogenous 17 kDa α-syn monomers. CD is synthesized as a prepropeptide (53 kDa); the signal peptide is cleaved upon CD insertion into the endoplasmic reticulum (47 kDa). The CD zymogen is then activated in the acidic lysosomal environment to produce the 32 and 14 kDa products [13,47]. d. Enhanced CD expression reduces A53T and A30P mutant α-syn-induced cell death, but does not reduce Y125A mutant α-syn-, 10 μM chloroquine-, or 2 μM staurosporine-induced cell death. For a-d, *p < 0.05 compared to control (CTL); †*p *< 0.05 compared to otherwise identical transfection except without CD. n = 3 transfection for each experimental conditions. Student t-test was used. e. Increased protein levels correlate with transfection of respective cDNAs. SHSY5Y cells were transfected with control vector, or respective cDNA in each lane. The respective antibodies used for the immunoblots are at the left side of the gel images. Mutated α-syn-GFP were produced. CD transfection did not produce significant truncation intermediate products on the mutated α-syn-GFP, suggesting that the protection against A53T and A30P may be via alteration of their intracellular targeting rather than direct cleavage. CD 53 kDa and 47 kDa precursors and the 32 kDa mature product are shown. Actin immunoblot was used as a loading control.

Excessive α-syn induces neuron death in cell cultures, and in a variety of genetic and viral delivery based animal models [42-45]. To examine the potential of elevating CD level as a means to reduce α-syn-induced cell death, we transfected human neuroblastoma SHSY5Y cells with α-syn-GFP, in the absence or presence of increased *CD *expression (Fig. 5a). Similar to previous studies of α-syn overexpression in yeast, worms and rat neurons[44], we found that overexpression of α-syn-GFP induced significant cell death in SHSY5Y cells. Co-transfection of human CD provided significant protection against α-syn-GFP overexpression-induced cell death (Fig. 5a). Quantitative real-time RT-PCR confirmed the up-regulation of α-syn mRNA by α-syn transfection, and lack of effect of CD transfection on α-syn mRNA level (Fig. 5b). Furthermore, co-expression of CD with α-syn-GFP in human neuroblastoma SHSY5Y cells produced a cleavage product of α-syn-GFP and reduced endogenous monomeric 17 kDa α-syn (Fig. 5c).

### α-syn point mutation at the major CD cleavage site results in resistance to CD neuroprotection

α-syn is rich in hydrophobic amino acids (52%) and is natively unfolded. CD has a known specificity in recognizing hydrophobic residues[11]. Although α-syn contains many putative CD cleavage sites, the main cleavage occurs at Y125[11]. We found that CD is also protective against PD-causing mutant α-syn-GFP induced-cell death in SHSY5Y cells (Fig. 5d). In contrast, mutating α-syn-GFP at the putative CD cleavage site Y125[11] results in an α-syn mutant that induces cell death that resists neuroprotection by elevated CD (Fig. 5d). Furthermore, CD is ineffective at attenuating chloroquine- or staurosporine-induced cell death (Fig. 5d). Western blot analyses confirmed the up-regulation of transfected genes (Fig. 5e).

### Overexpression of CD is neuroprotective in *C. elegans*

**Figure 6:**
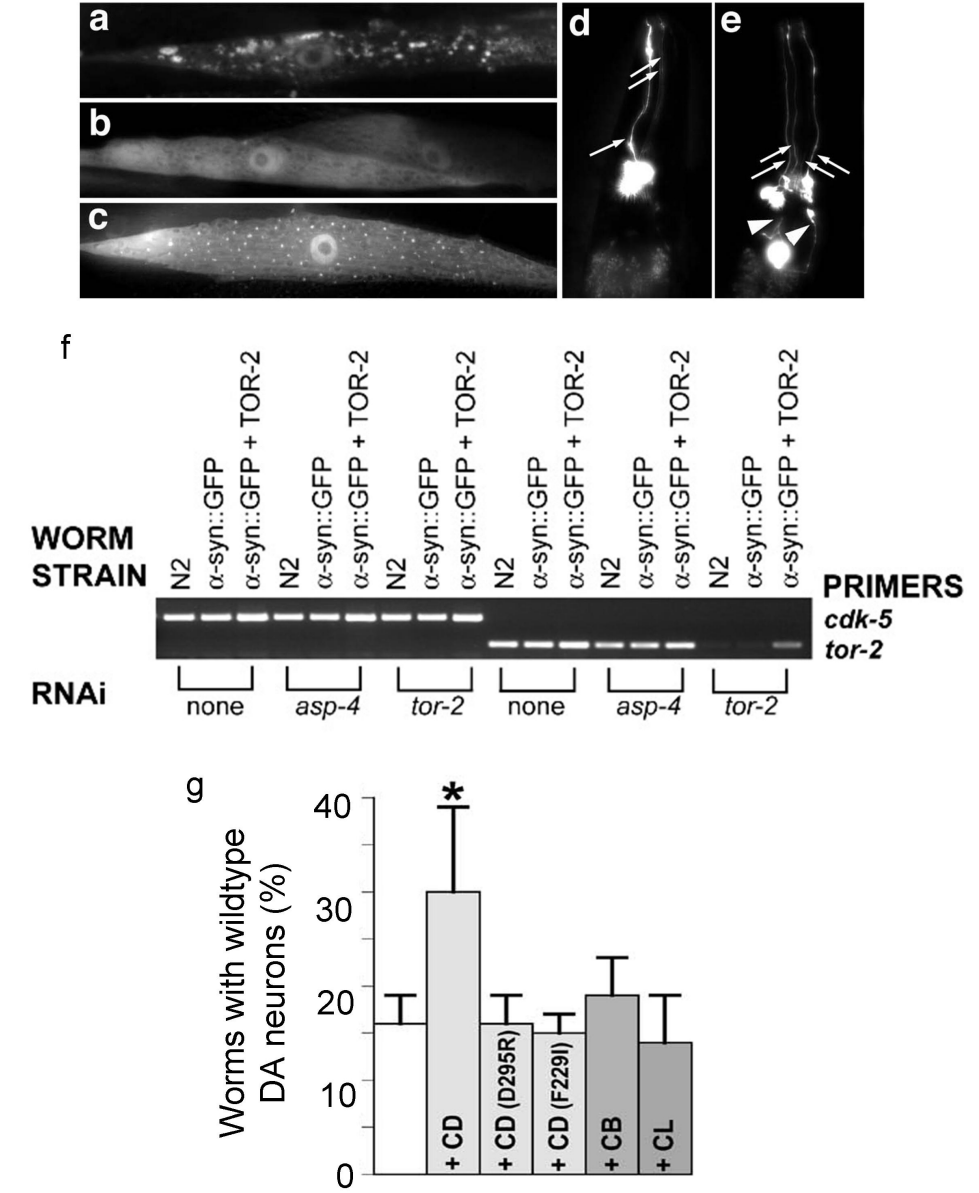
**Increased CD expression reduces α-syn-toxicity in *C. elegans***. a. RNAi knockdown of a *C. elegans Ctsd *ortholog worsens aggregation of human α-syn in vivo. Isogenic worm strain expressing α-syn::GFP alone (a) or with TOR-2 (b), in body wall muscle cells of *C. elegans*. The presence of TOR-2, a protein with chaperone activity, attenuates the misfolded α-syn protein (b). When worms expressing α-syn::GFP + TOR-2 are exposed to *CD *RNAi, the misfolded α-syn::GFP returns (c). d-f. Overexpression of CD protects DA neurons from α-syn-induced degeneration. Worm DA neurons degenerate as animals age. At the 7 d stage, most worms are missing anterior DA neurons of the CEP (cephalic) and/or ADE (anterior deirid) classes. d. Note the presence of 3 of 4 CEP DA neurons (arrows) and the absence of the 2 ADE neurons. e. Overexpression of CD protects worms from neurodegeneration whereby worms display all 4 CEP (arrows) and both ADE (arrowheads) neurons. f. RNAi knock down of *asp-4 *does not reduce *tor-2 *expression level. Semi-quantitative RT-PCR was performed by using primers to amplify *cdk-*5 (loading control) and *tor-2*. For all strains analyzed, normal (non-RNAi) condition was used as a negative control, and *tor-2 *RNAi was used as a positive control.g. The percentage of worms exhibiting the wildtype neuronal complement of all 6 anterior DA neurons (30%) was significantly greater than animals without CD overexpression (15%). CD mutants (D295R and F229I), CB and CL, in transgenic worms overexpressing human cDNAs encoding these mutated CD or the representative lysosomal cysteine proteases, do not have the same effect as the wildtype CD in reducing α-syn toxicity. *p < 0.001 compared to α-syn alone, by Fisher Exact Test.

To further investigate the role of CD activity in α-syn clearance in vivo, we generated transgenic *C. elegans *expressing a human α-syn and GFP fusion protein in body wall muscle cells. In these worms, human α-syn::GFP forms aggregates as worms develop and age (Fig. 6a). As in mammalian cells[46], co-expression of the worm TOR-2 protein chaperone ameliorated the formation of α-syn::GFP aggregates (Fig. 6b). Importantly, this established a genetic background within which enhancement of α-syn aggregation could be more readily visualized by RNA interference (RNAi). Using bacterial RNAi feeding to specifically target the *C. elegans *ortholog of *Ctsd*, we knocked down *Ctsd *in α-syn::GFP + TOR-2 transgenic worms. RNAi targeting of *Ctsd *led to a return of fluorescent aggregates over time (Fig. 6c), and RNAi of *Ctsd *did not affect *tor-2 *mRNA levels as examined by real-time RT-PCR (Fig. 6f). Thus, *Ctsd *deficiency led to α-syn aggregation in both mice and worms.

We further investigated whether human CD attenuates the loss of dopaminergic neurons in a *C. elegans *model of α-syn-induced neurodegeneration[44]. Overexpression of α-syn led to DA neuron death, as evidenced by the finding that only 16% of 7 d old adult α-syn expressing worms (n = 270 worms analyzed) displayed normal numbers of DA neurons (Fig. 6d, g). In contrast, co-overexpression of human CD significantly protected against DA neurodegeneration, since 30% of same-staged animals (n = 270) exhibited wildtype DA neurons (Fig. 6e, g; *p *< 0.001, Fisher Exact Test). Overexpression of enzymatic mutants of CD (D295R and F229I) [16,47], or related human cathepsin gene products, CB or CL, did not attenuate DA neuron death in this *in vivo *assay (Fig. 6g), thereby suggesting a specific role of CD in neuroprotection against α-syn-induced DA cell death, as well as an essential role of CD enzymatic activity in this neuroprotection.

## Discussion

Our results demonstrate that CD deficiency leads to significant accumulation of α-syn that is concentrated in neuronal cell bodies, despite the up-regulation of multiple lysosomal cathepsin genes. α-synucleinopathy arises from CD dysfunction, either directly due to deficient proteolytic cleavage, and/or indirectly as a consequence of compromised proteasome activity. Most significantly, we further demonstrated that CD overexpression protects neurons from α-syn aggregation and α-syn-induced death both in mammalian cells and in *C. elegans*. These findings suggest that targeted CD therapy may inhibit or reverse LB disease-associated neuropathology.

The α-syn accumulations in *Ctsd*-/- brains are neuronal and perinuclear. Although *Ctsd*-/- brain extracts exhibited increased immunoreactive protein species recognized by the anti-phospho-α-syn antibody that specifically recognizes human LB[36], α-syn accumulations in *Ctsd*-/- brains could not be detected by this antibody that specifically recognizes human LB despite that they were successfully used in our hands to demonstrate LB-specific staining of human PD specimen (data not shown). Only 5% of neurons exhibited intense immunoreactivity for both α-syn and ubiquitin suggesting their accumulation, an observation in accordance with that only a small fraction of α-syn is ubiquitinated in human LB [33-36]. α-syn accumulations are adjacent to but not overlapping with autophagosomal or lysosomal markers, suggesting an attenuation of these complexes and organelles in encircling accumulated α-syn in the *Ctsd*-/- brains. Where α-syn unfolds and forms oligomers, and whether CD reduces a specific sub-population of α-syn protofibrils or other small assemblies of α-syn require further investigation.

α-syn gene triplication is causative in a subset of PD, which suggests that increasing α-syn gene dosage promotes DA neuron death in these patients[1]. Overproduction of α-syn via AAV delivery induces DA neuron death in rodents[45]. CD deficiency induced by aging, genetic polymorphism, or environmental toxins may predispose animals to an earlier onset of spontaneous neuron death, or exacerbate neurotoxin or α-syn overproduction-induced DA neuron loss, in comparison to wildtype control mice. Furthermore, although *Ctsd*-/- mice die approximately p26 without apparent tau or beta amyloid accumulation in the brain (data not shown), we cannot exclude the possibility that partial loss of CD may also affect tau or beta amyloid clearance.

We found significant down-regulation of proteasome activities in *Ctsd*-/- brains. The reduction of proteasome activity may be due to reduced expression of UPS regulators and proteasome subunits, inactivating post-translational modification of these proteins, or specific inhibitory signaling by the accumulated ubiquitinated proteins. Crosstalk between protein degradation pathways is potentially important in neurodegenerative disease pathogenesis since inhibition of proteasomes up-regulates lysosomal enzymes[48], and enhancing proteasome function alleviates autophagy defects[49]. Furthermore, α-syn overexpression in mammalian cells affects proteasome activity, macroautophagy as well as lysosomal functions[50]. Lysosomal storage disorders and lysosomal cysteine protease inhibitor E64 treatment were previously shown to down-regulate UCHL1[51], however, such is not the case in *Ctsd*-/- brains in our study, indicating a distinct mechanism of CD deficiency-induced proteasome dysfunction. GAPDH accumulation may be a consequence of impaired CMA[3], because *Ctsd*-/- cells are compromised in lysosomal function[3]. Alternatively, GAPDH elevation may represent an ultimately futile compensatory signal that elevates macroautophagy initiation as previously suggested in HeLa cells[52]. Additionally, GAPDH elevation may promote the formation of protein inclusions [53,54].

The overproduction of α-syn has been shown to cause cell death in many studies through a variety of mechanisms, including a compromise in synaptic vesicle function, the induction of ER stress, Golgi fragmentation, mitochondrial dysfunctions and proteasome dysfunction [44,55-58]. However, cell death in these studies required massive overproduction of α-syn. Even in these conditions, the challenge has been to determine whether the intermediate species of α-syn are the most toxic to neurons. It remains possible that the α-syn aggregates in sporadic PD are not causative to DA neuron death, but rather a compensatory activity to attenuate DA neuron death, or a mere bystander effect in DA neuron death. The comparison of different mutant forms of α-syn in their propensity in forming fibrils and their toxicity was carried out in yeast and the rate of fibrillization did not positively correlate with toxicity[59]. α-syn can interact with many other proteins[60], but whether these interactions elicit cytoprotective versus death-inducing effects remains to be elucidated. Other observations suggest that LB formation may be a mechanism to sequester toxic α-syn species and thus exert a protective function [61-63]. Our observation that α-syn accumulates in *Ctsd*-/- brains does not distinguish its role in promoting or attenuating death in *Ctsd*-/- brains in vivo. We did not observe neurons with concurrent activation of caspase-3 and α-syn accumulation (Fig. 2). This may indicate that α-syn accumulation and capase-3 activation are with different time course. Alternatively, our observation may suggest that? certain forms of α-syn accumulation induce non-apoptotic cell death, or even compensatory neuroprotection.

α-syn knockout mice are resistant to MPTP-induced DA neuron death [64-66]. Reduction of α-syn is neuroprotective in multiple animal models [67,68]. Regardless of the precise mechanism for CD deficiency induced α-synucleinopathy, our finding that enhanced expression of CD is neuroprotective against α-syn aggregation and toxicity, both in worms and in mammals, provides a basis for future investigation of whether delivery of CD to the central nervous system in animal models of LB diseases can help alleviate protein aggregation and toxicity, and thereby serve as a previously unexplored target for disease therapy.

## Methods

### Mice

We genotyped littermates from *Ctsd*+/- breeding. We used *Ctsd*+/+, *Ctsd*+/- and *Ctsd*-/- littermates on C57BL6 background for all experiments.

### Immunohistochemistry

Brains were placed in Bouin's fixative overnight at 4°C followed by paraffin embedding. 5 μm thick sections were used for immunohistochemical studies. The following antibodies were used: mouse anti-NeuN (Chemicon), mouse anti-α-syn (BD Transduction Lab), sheep anti-α-syn (Chemicon), goat anti-CD (Santa Cruz), mouse anti-GFAP (Chemicon), rabbit anti-Ub (Dako), mouse anti-Ub (FK2, Biomol), mouse anti-Ub (Chemicon, clone Ubi-1), mouse anti-Ub (Zymed), mouse anti-synaptophysin (Chemicon), goat anti-cathepsin B (Santa Cruz), rabbit anti-active caspase 3 (Chemicon), and rabbit-anti-GAPDH (Cell Signaling). Horseradish peroxidase conjugated donkey derived secondary antibodies were used at 1:2000 (Jackson ImmunoResearch). The sections were then incubated with tyramide signal amplification (TSA) plus detection solution with Cy3 or fluorescein tyramide according to the manufacturer's instruction (PerkinElmer Life Sciences), followed by bisbenzimide staining of DNA. For chromogenic immunohistochemistry, we incubated the sections with a biotinylated secondary antibody (Vector Laboratories) followed by ABC reagent (ABC kit, Vector Laboratories). We visualized the immunoreaction by treating the sections in 0.05% diaminobenzidine (DAB) with hydrogen peroxide. The images were taken using a Leica TCS SP5 confocal microscope or a Zeiss Axiocam CCD camera on a 100 W Axioscope bright field and fluorescence microscope.

### α-syn aggregation assay

We used the in vitro system developed by McLean and colleagues in which an α-syn-green fluorescent protein (GFP) fusion protein (α-syn-GFP) becomes truncated at the C-terminus, cleaving off GFP, to form visible aggregates in cells when co-expressed with synphilin[41]. We transfected H4 neuroglioma cells with α-syn-GFP and synphilin, and either the empty vector pcDNA3.1 or CD. 24 h after transfection, cells were fixed and stained with a mouse monoclonal antibody against α-syn (1:1000; BD transduction), and a secondary Alexa 488-conjugated goat anti-mouse antibody (1:500; Jackson ImmunoResearch). An observer blind to the transfection conditions scored neurons as positive or negative for α-syn aggregates visible with a 20X objective under a fluorescent microscope. Three independent experiments were carried out with 4 replicates per experiment. Student t-test was used to compare transfection with empty vector versus transfection with CD.

### Cell culture and transfection

The human neuroblastoma SHSY5Y cells were transfected in triplicate by vector alone, a-syn-GFP, pCMV-CD, or co-transfected by a-syn-GFP (or A53T, A30P, Y125A mutant a-syn) and pCMV-CD (or mutant CD) by Amaxa method as described by the vendor. Transfection efficiency was 80% as assessed by cells with or without GFP. 72 h after transfection, cells were harvested. For chloroquine (10 mM) treatment, the chemicals were added 48 h after transfection, cells were harvested 42 h later. Live cells were counted by trypan blue exclusion. Relative cell survival was calculated as number of live cells after transfection by pCMV-CD and/or a-syn-GFP divided by live cells after transfection by vector alone. Elevated expression of a-syn-GFP or cathepsins was confirmed by western blot analyses using whole cell extracts.

### Western blot

We homogenized wildtype and *Ctsd*-/- cortex (n ≥ 3 each genotype) in 10 volumes of ice-cold lyses buffer (50 mM Tris-HCl pH 7.4, 175 mM NaCl, 5 mM EDTA), sonicated for 10 sec, add TritonX-100 to 1% and incubated for 30 min on ice. We then centrifuged homogenates at 15,000 *g *for 15 min at 4°C to separate supernatants (fractions soluble in 1% TritonX-100) and pellets (TritonX-100-insoluble fractions)[43]. Pellets were resuspended in lyses buffer containing 2% SDS. Western blotting for each sample was done at least twice. The antibodies used were described in Immunohistochemistry.

### Quantitative PCR

Total brain RNA was isolated from p25 mice using RNA-STAT60 (Tel-Test, Friendswood, TX). Total RNA (2 μg) was then reverse transcribed using Applied Biosystems GeneAmp Gold RNA PCR Reagent Kit (Foster City, CA). Real-time PCR reactions were setup in duplicate using TaqMan gene assays and amplified in an Applied Biosystems Step-One instrument. ΔΔCCT curves were generated using 18S TaqMan gene assays as internal standards. Quantitative PCR results are shown as standard deviation of from 3 different amplifications from RNA reverse transcribed from 3 different animals. Individual gene assay kits were purchased from Applied Biosystems for each of the RNAs analyzed. Paired t-tests were conducted on RQ (relative quantity) values for each group to determine significance.

### Proteasome activity assays

We analyzed the proteasome activities using the TritonX-100-soluble fractions. The assay buffer consists of 50 mM Tris (pH7.5), 2.5 mM EGTA, 20% glycerol, 1 mM DTT, 0.05% NP-40, 50 μM substrate. Lactacystin was used at a final concentration of 10 μM to block proteasome activities as negative controls. Fluorescence was read at 5 min intervals for 2 h, at an excitation wavelength of 380 nm and an emission wavelength of 460 nM. Assays were done in triplicate, each using n ≥ 3 mice per genotype.

### *C. elegans *experiments

Nematodes were maintained following standard procedures[69]. Worms expressing α-syn alone UA49 [*baInl2*; *P*_*unc*-54_::*α-syn*::*gfp*, *rol-6 (su1006)*] or with *tor-2 *[UA50; *baInl3*; *P*_*unc-54*_::*α-syn*::*gfp*, *P*_*unc-54*_::*tor-2, rol-6 (su1006)*] were created, integrated into the genome to generate an isogenic line, and out-crossed four times. We used the worm line that overexpresses TOR-2 protein (a worm homolog of human torsinA) and a-syn fused to GFP in the body wall muscle cells because these cells are much larger than neurons for detecting a-syn aggregation. Moreover, C. elegans dopaminergic neurons have been shown to be refractory to RNAi. Using this isogenic line, we knocked down the worm Ctsd ortholog by RNAi, and scored for the return of a-syn aggregates over the course of development and aging.

RNAi was performed by bacterial feeding as described[70] with the following modification. A *Ctsd*-specific RNAi feeding clone targeting a distinct portion of the *C. elegans *open reading frame [R12H7.2 (*asp-4*); e-value = 1.8e-108] with highest homology to human *Ctsd *(Geneservice) was grown for 14 h in LB broth with 100 μg/ml ampicillin and seeded onto NGM agar plates containing 1 mM isopropyl β-D-thiogalactoside. After 4 h incubation at 25°C to dry the plates, five gravid adults were then placed onto the corresponding RNAi plates and allowed to lay eggs for 9 h; the resulting age-synchronized worms were analyzed at the indicated stage. RNAi knockdown was performed in duplicate sets of animals and enhancement α-syn misfolding was scored as positive if at least 80% of worms displayed an increased quantity and size of α-syn::GFP aggregates. For each trial, 20 worms were transferred onto a 2% agarose pad, immobilized with 2 mM levamisole, and analyzed using Nikon Eclipse E800 epifluorescence microscope equipped with Endow GFP HYQ filter cube (Chroma Technology). Images were captured with a Cool Snap CCD camera (Photometrics) driven by MetaMorph software (Universal Imaging).

### Quantitative PCR from C. elegans

The procedure for total RNA isolation, cDNA preparation, and semi-quantitative RT-PCR was described previously[71]. The following primers were used for the PCR: *cdk-5 *Primer 1: 5' ggg-gat-gat-gag-ggt-gtt-cca-agc 3'; Primer 2: 5' ggc-gac-cgg-cat-ttg-aga-tct-ctg-c 3'. *tor-2 *Primer 1: 5' caa-tta-tca-tgc-gtt-ata-caa-ag 3'; Primer 2: 5' cat-tcc-act-tcg-ata-agt-att-g 3'. *cdk-5 *Primer 1: 5' ggg-gat-gat-gag-ggt-gtt-cca-agc 3'; Primer 2: 5' ggc-gac-cgg-cat-ttg-aga-tct-ctg-c 3'. *tor-2 *Primer 1: 5' caa-tta-tca-tgc-gtt-ata-caa-ag 3'; Primer 2: 5' cat-tcc-act-tcg-ata-agt-att-g 3'.

For the DA neurodegeneration analysis, strain UA54 [*baEx45*; *P*_*dat-1*_::*α-syn, P*_*dat-1*_::*gfp, P*_*dat-1*_::*CD*,*rol-6 (su1006)*], UA90 [*baEx69*; *P*_*dat-1*_::*α-syn, P*_*dat-1*_::*gfp, P*_*dat-1*_::*CD D295R*,*rol-6 (su1006)*], UA91 [*baEx70*; *P*_*dat-1*_::*α-syn, P*_*dat-1*_::*gfp, P*_*dat-1*_::*CD F229I*,*rol-6 (su1006)*], UA53 [*baEx44*; *P*_*dat-1*_::*α-syn, P*_*dat-1*_::*gfp, P*_*dat-1*_::*CB*,*rol-6 (su1006)*], and UA55 [*baEx46*; *P*_*dat-1*_::*α-syn, P*_*dat-1*_::*gfp, P*_*dat-1*_::*CL*,*rol-6 (su1006)*] were generated by injecting 50 μg/ml of expression plasmid containing the human cathepsin cDNA and 50 μg/ml of *rol-6 *into an integrated line of UA44 [*baInl1*; *P*_*dat-1*_::*α-syn, P*_*dat-1*_::*gfp[72]*]. Three stable lines were randomly selected for neurodegeneration analysis. The 6 anterior DA neurons (4 CEP and 2 ADE neurons) of 30 animals/trial were examined for neurodegeneration when the animals were 7 days old. 90 animals from each of three CD (or CD D295R, CD F229I, CB, and CL) transgenic lines were analyzed (3 lines × 3 trials of 30 animals/trial = 270 total animals scored). Worms displaying at least one degenerative change (dendrite, axon, or cell body loss) were scored as exhibiting degenerating neurons as previously reported [44;72].

## Abbreviations

α-syn: alpha-synuclein; PD: Parkinson's disease; CD: Cathepsin D; GFP: green fluorescent protein; RNAi: RNA interference; UPS: ubiquitin-proteasome system.

## Competing interests

KAC and GAC are scientific advisors to QRxPharma, Ltd. from whom they receive monetary compensation and a sponsored research agreement. The other authors have declared that no conflict of interest exists.

## Authors' contributions

LQ performed the majority of the experiments of immunohistochemistry, western blot analyses, proteasome activity assays, and cell culture studies. SH, KAC and GAC contributed to all the findings in the worm. TAY contributed the α-syn aggregation assay in H4 cells. SW contributed the real-time PCR. ZLX, RP, LDS, DC, QL, SC and LS assisted with various experiments. YU provided the LC3 antibody. TI provided the anti-phospho-α-syn antibody. YZ constructed mammalian expression plasmids for GFP-α-syn. LP and YML constructed the mammalian expression plasmid for CD. KCW, JJS and KAR contributed the original CD mutant mice, brain sections and extracts for our initial findings, helped with setting up western and immunohistochemistry techniques, and provided discussions on autophagy and CD. KAR was essential for identification of the α-syn aggregates after immunostaining and provided neuropathology expertise, discussions, and assistance in writing the manuscript. DGS provided discussions and expertise in PD, α-syn metabolism, and confocal microscopy. JZ directed the project and wrote the manuscript.
